# Proteomic signatures predict preeclampsia in individual cohorts but not across cohorts – implications for clinical biomarker studies

**DOI:** 10.1080/14767058.2021.1888915

**Published:** 2021-03-02

**Authors:** Mohammad S. Ghaemi, Adi L. Tarca, Roberto Romero, Natalie Stanley, Ramin Fallahzadeh, Athena Tanada, Anthony Culos, Kazuo Ando, Xiaoyuan Han, Yair J. Blumenfeld, Maurice L. Druzin, Yasser Y. El-Sayed, Ronald S. Gibbs, Virginia D. Winn, Kevin Contrepois, Xuefeng B. Ling, Ronald J. Wong, Gary M. Shaw, David K. Stevenson, Brice Gaudilliere, Nima Aghaeepour, Martin S. Angst

**Affiliations:** a Department of Anesthesiology, Perioperative and Pain Medicine, Stanford University School of Medicine, Stanford, CA, USA; b Department of Obstetrics and Gynecology, Wayne State University School of Medicine, Detroit, MI, USA; c Department of Obstetrics and Gynecology, University of Michigan, Ann Arbor, MI, USA; d Department of Obstetrics and Gynecology, Stanford University School of Medicine, Stanford, CA, USA; e Department of Genetics, Stanford University School of Medicine, Stanford, CA, USA; f Department of Surgery, Stanford University School of Medicine, Stanford, CA, USA; g Department of Pediatrics, Stanford University School of Medicine, Stanford, CA, USA; h Department of Biomedical Data Sciences, Stanford University School of Medicine, Stanford, CA, USA

**Keywords:** Biomarker, gestational age, preeclampsia, pregnancy, proteomics

## Abstract

**Background:**

Early identification of pregnant women at risk for preeclampsia (PE) is important, as it will enable targeted interventions ahead of clinical manifestations. The quantitative analyses of plasma proteins feature prominently among molecular approaches used for risk prediction. However, derivation of protein signatures of sufficient predictive power has been challenging. The recent availability of platforms simultaneously assessing over 1000 plasma proteins offers broad examinations of the plasma proteome, which may enable the extraction of proteomic signatures with improved prognostic performance in prenatal care.

**Objective:**

The primary aim of this study was to examine the generalizability of proteomic signatures predictive of PE in two cohorts of pregnant women whose plasma proteome was interrogated with the same highly multiplexed platform. Establishing generalizability, or lack thereof, is critical to devise strategies facilitating the development of clinically useful predictive tests. A second aim was to examine the generalizability of protein signatures predictive of gestational age (GA) in uncomplicated pregnancies in the same cohorts to contrast physiological and pathological pregnancy outcomes.

**Study design:**

Serial blood samples were collected during the first, second, and third trimesters in 18 women who developed PE and 18 women with uncomplicated pregnancies (Stanford cohort). The second cohort (Detroit), used for comparative analysis, consisted of 76 women with PE and 90 women with uncomplicated pregnancies. Multivariate analyses were applied to infer predictive and cohort-specific proteomic models, which were then tested in the alternate cohort. Gene ontology (GO) analysis was performed to identify biological processes that were over-represented among top-ranked proteins associated with PE.

**Results:**

The model derived in the Stanford cohort was highly significant (*p* = 3.9E–15) and predictive (AUC = 0.96), but failed validation in the Detroit cohort (*p* = 9.7E–01, AUC = 0.50). Similarly, the model derived in the Detroit cohort was highly significant (*p* = 1.0E–21, AUC = 0.73), but failed validation in the Stanford cohort (*p* = 7.3E–02, AUC = 0.60). By contrast, proteomic models predicting GA were readily validated across the Stanford (*p* = 1.1E–454, *R* = 0.92) and Detroit cohorts (*p* = 1.1.E–92, *R* = 0.92) indicating that the proteomic assay performed well enough to infer a generalizable model across studied cohorts, which makes it less likely that technical aspects of the assay, including batch effects, accounted for observed differences.

**Conclusions:**

Results point to a broader issue relevant for proteomic and other omic discovery studies in patient cohorts suffering from a clinical syndrome, such as PE, driven by heterogeneous pathophysiologies. While novel technologies including highly multiplex proteomic arrays and adapted computational algorithms allow for novel discoveries for a particular study cohort, they may not readily generalize across cohorts. A likely reason is that the prevalence of pathophysiologic processes leading up to the “same” clinical syndrome can be distributed differently in different and smaller-sized cohorts. Signatures derived in individual cohorts may simply capture different facets of the spectrum of pathophysiologic processes driving a syndrome. Our findings have important implications for the design of omic studies of a syndrome like PE. They highlight the need for performing such studies in diverse and well-phenotyped patient populations that are large enough to characterize subsets of patients with shared pathophysiologies to then derive subset-specific signatures of sufficient predictive power.

## Introduction

Preeclampsia (PE) is a multisystem disorder of pregnancy defined by the new onset of arterial hypertension and proteinuria after 20 weeks of gestation. It is a leading cause of maternal and perinatal morbidities affecting 2–5% of women worldwide [1,2]. Despite recent advances in our understanding of the pathophysiologies that drive PE, we still lack molecular biomarkers of sufficient power for early risk prediction ahead of clinical manifestations [3,4].

The quantitative analyses of plasma proteins for early risk prediction have received significant attention [5-8]. For example, soluble fms-like tyrosine kinase 1 (sFlt-1) and placental growth factor (PIGF) are useful in identifying women who will not develop PE [9,10]. However, derivation of biosignatures with high positive predictive power to reliably identify women at risk for developing PE remains a high priority. The inclusion of additional biomarkers has been a suggested strategy to enhance positive predictive power [8,10].

The derivation of predictive models of PE has largely been anchored in our current understanding of its underlying pathophysiologies. For example, the SCOPE study examined 47 serum proteins based on their associations with PE and their biological roles in placentation and in cellular mechanisms implicated in its pathogenesis [6]. However, derived prediction models were of limited power [11]. An alternative and more exploratory approach has recently been enabled by the availability of highly multiplexed proteomic arrays that simultaneously measure over 1000 plasma proteins in a single blood sample [12-14].

The primary aim of this study was to derive separate proteomic signatures predicting the risk of PE in two independent cohorts (Stanford and Detroit) of pregnant women using the same highly multiplexed proteomic arrays and multivariate analysis approaches, and then test their generalizability across cohorts. A secondary aim was to demonstrate generalizability of proteomic signatures predicting a physiological, rather than a pathophysiological outcome to provide biological evidence for the adequate technical performance of the proteomic platforms across both study cohorts.

## Materials and methods

### Study design

Pregnant women presenting to the Obstetrics Clinics of the Lucile Packard Children’s Hospital at Stanford University were invited to participate in a prospective cohort study sponsored by the March of Dimes Prematurity Research Center to examine an array of environmental and biological factors associated with uncomplicated and pathological pregnancies [15,16]. All women were eligible if they were at least 18 years of age and in their first trimester of pregnancy. Blood samples were obtained during the first (7–14 weeks), second (15–20 weeks), and third (24–32 weeks) trimesters of pregnancy. In two subsets of women (18 with early- or late-onset PE and 18 with uncomplicated term pregnancies) with an equal number of serial blood specimens (2–3 per women; 98 total) detailed proteomic analyses were performed. The control group represented a random selection from the general population seen at the Obstetrics Clinics. The number of women included in the study is explained by the relatively low number of women who developed PE during the observation period including over 300 women. The study was approved by the Institutional Review Board of Stanford University School of Medicine and all participants provided written informed consent.

### Gestational age (GA)

GA was determined by best obstetrical estimate as recommended by the ACOG [17].

### PE diagnostic criteria

PE and its severity were diagnosed based on the criteria recommended by the Task Force of the ACOG on Hypertension in Pregnancy [18].

### Plasma samples

Blood was collected into EDTA tubes, placed in ice, and double-spun. Plasma was stored at −80 °C and all processing was completed within 60 min of collection.

### Proteomic assays

All analyses were performed in randomly allocated samples by SomaLogic, Inc. (Boulder, CO) using a highly multiplex aptamer-based platform [19,20]. The assay quantifies relative concentrations of proteins over a wide dynamic range (>8 log) using chemically modified aptamers with slow off-rate kinetics (SOMAmer reagents). Each SOMAmer reagent is a unique, high-affinity, single-strand DNA endowed with functional groups mimicking amino acid side chains. Nucleotide signals are quantified using relative florescence on microarrays. The assay has a historic median intra- and inter-run coefficient of variation of about 5%, and median lower and upper limits of quantification of 3.0 pM and 1.5 nM [19].

### Derivation of a proteomic model predicting PE in Stanford cohort

For a matrix *X* containing all proteins (features), and a binary vector of PE *Y*, a multivariate logistic regression model with penalization (LASSO) was developed [21]. The equations are provided in the supplement.

A cross-validation procedure tested for the generalizability of the multivariate models to previously unseen samples. To account for interdependencies between samples from the same woman, for each cross-validation iteration, all samples corresponding to the entire time series from one woman were excluded from the training cohort used to build the model. The resulting model was then used for estimating PE for the excluded women. The procedure was repeated until an estimation of PE was obtained for all sampling times points and each woman.

### Validation of a proteomic model predicting PE in Detroit cohorts

Using fixed parameters, the validity of the model derived in the Stanford cohort was tested using proteomic data from the Detroit cohort consisting of 90 women with uncomplicated pregnancies and 76 women with late-onset PE [12]. The analysis was then reversed. A proteomic model predicting PE was derived in the Detroit cohort, and then tested in the Stanford cohort. Data on the same 1116 proteins were available to infer a predictive model in both cohorts.

### Derivation and validation of proteomic models predicting GA as an exemplary physiological outcome

A multivariate linear regression model with penalization (LASSO) was used to derive a model predicting GA at the time of blood collection in women with uncomplicated pregnancies. Proteomic models predicting GA were independently derived in the Stanford and the Detroit cohort and then tested for validity in the alternate cohort.

### Data transformation and controlling for batch effects

A variety of transformation approaches including log transformation were examined. Similarly, batch effects between the two cohorts were examined with SVA/ComBat [22]. None of these approaches improved predictive power of derived models as judged by respective areas under the curve (AUCs, data not shown). Consequently, data were not transformed for the final analysis.

### Consideration of GA at the time of sampling when predicting PE

Various analyses were performed to examine whether integrating GA at the time of sampling into the model would improve predictive power. Approaches included consideration of trimesters, using LASSO with a non-linear kernel, using a local LASSO (multiple fits over a sliding window on GA followed by smoothing), and using a varying coefficient approach that could smoothly change the model over GA [23]. None of these approaches improved the generalizability between the two datasets as judged by respective AUCs.

### Univariate analyses

Univariate analyses of protein abundance were performed using mixed effect models with GA at time of sampling as a fixed effect and allowing for a random effect for each woman [24]. This approach accounts for multiple correlated measurements from the same woman.

Unpaired *t*-test was used for continuous data and Chi-square test was used for categorical data to examine group differences of demographic and pregnancy data. Unadjusted *p* values <5E–02 are reported. Adjusting for multiple comparisons in our analysis, a *p* value <3.8E–03 is required to indicate statistical significance.

### Correlation network

Spearman’s correlation analyses using R were performed between all pairs of proteins. The correlation network was built in data collected from all women of the Stanford cohort. The graphical representation of the correlation network shows edges for significant correlations between two respective proteins (*p* < 1.0E–37, Bonferroni’s corrected and further adjusted for sparse representation). The graph layout was calculated using the t-SNE algorithm [25].

### GO enrichment analysis

Gene ontology (GO) analysis affords a more integrative examination of the proteomic data, which can point to biological processes that underlie PE. The GO enrichment analysis was performed to identify GO terms that were over-represented among top-ranked gene products associated with PE. Enrichment analysis was performed using the “topGO” R package and Fisher’s exact test [26,27].

## Results

All raw data are available at https://figshare.com/articles/Proteomic_Models_in_Preeclampsia/7962998.

### Study subjects

Patient and pertinent pregnancy data are provided in Table 1. The body mass index in women with PE was higher in the Stanford (*p* = 6.0E–03) and the Detroit cohort (*p* = 2.4E–02) when compared to women with uncomplicated pregnancies. Gravity was higher in women with uncomplicated pregnancy than in women with PE in the Detroit cohort (*p* = 3.3E–02). The fraction of nulliparous women, GA at delivery for late PE, and the percentage of severe PE were similar in the Stanford and Detroit cohorts. Women in the Detroit cohort were younger (*p* < 1.0E–03), had a higher body mass index (*p* < 1.0E–03), and higher gravidity (*p* = 1.0E–02) compared with the Stanford cohort. Sharply contrasting with the Stanford cohort, was the racial distribution in the Detroit cohort which consisted of 94% African Americans (*p* < 1.0E–03).

### Assay quality control

All quality metrics for the proteomic assay were met with plate scale factors of 1.24 and 1.46, and SOMAmer calibration factors <0.4 for 95% of SOMAmers. The median coefficient of variation was 4.1%.

### GA

All women had ultrasound exams during the first trimester of pregnancy. In 35 women, GA was determined based on standard ultrasound metrics according to the guidelines of the ACOG [17]. In one woman, GA was known based on the date of *in vitro* fertilization.

### Proteomic models predicting PE in the Stanford and Detroit cohorts

Plasma proteins formed a correlation network that highlights the inter-connectivity of the proteomic changes over the course of a pregnancy (Supplementary Figure 1). Among the top 20 proteins included in each model best predicting PE in the Stanford or Detroit cohort, only leptin was shared.

### Validation of proteomic model predicting PE in the alternate cohort

The model derived in the Stanford cohort was highly significant (*p* = 3.9E–15) with excellent performance of the classifier separating PE from uncomplicated pregnancies (AUC = 0.96) (Supplementary Figure 2A). However, the model could not be validated in the Detroit cohort (*p* = 9.7E–01, AUC =0.50) (Supplementary Figure 2B). Similarly, the model derived in the Detroit cohort was highly significant with fair performance of the classifier (*p* = 1.0E–21, AUC = 0.73) (Supplementary Figure 2C), but failed validation in the Stanford cohort (*p* = 7.3E–02, AUC = 0.60) (Supplementary Figure 2D). These results did not change when excluding women with early-onset PE from the Stanford cohort.

### Proteomic model predicting a physiological (GA) outcome in the Stanford and Detroit cohorts

The rational for deriving proteomic models predicting GA in studied cohorts and confirm their generalizability across cohorts was to provide biological evidence supporting adequate technical performance of the proteomic platforms in both study cohorts (Supplementary Figure 3). The model derived in the Stanford cohort was highly significant (*p* = 1.1.E–101) with high predictive power (*R* = 0.93), and readily passed validation in the Detroit cohort (*p* = 1.1E–454, *R* = 0.91). Similarly, the model derived in the Detroit cohort was highly significant (*p* = 1.1.E–92) with high predictive power (*R* = 0.92), and readily passed validation in the Stanford cohort (*p* = 1.1E–488, *R* = 0.92).

### Individual proteins associated with PE and GA

**Figure 1.**
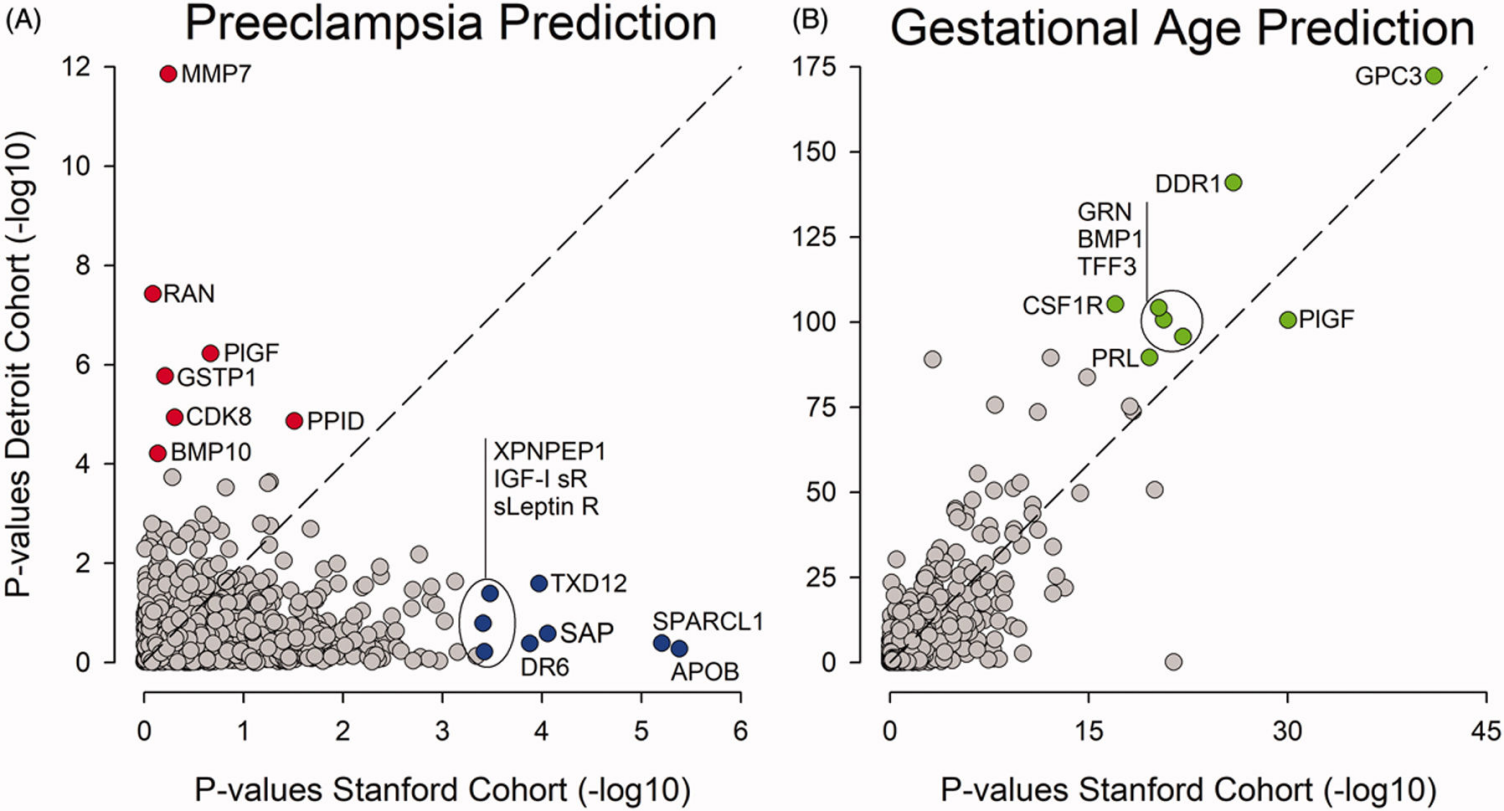
Highest-ranking proteins associated with PE differ between cohorts. (A) All 1116 proteins included in the analysis are plotted according to their respective *p* value when comparing women with PE to women with uncomplicated pregnancies in the Stanford (*x*-axis) and the Detroit (*y*-axis) cohorts. The highest-ranking proteins are not shared between the Stanford and the Detroit cohorts. (B) In contrast, the highest-ranking proteins predicting GA are shared between the Stanford and the Detroit cohorts . APOB: apolipoprotein; BMP1: bone morphogenetic protein 1; BMP10: bone morphogenetic protein 10; CDK8: cyclin-dependent kinase 8:cyclin-C complex; CSF1R: macrophage colony-stimulating factor 1 receptor; DDR1: discoidin domain receptor 1; DR6: tumor necrosis factor receptor superfamily member 21; GPC3: glypican-3; GRN: granulins; GSTP1: glutathione S-transferase P; IGI-I sR: insulin-like growth factor 1 receptor; sLeptin R: Leptin receptor; MMP7: matrilysin; PIGF: placenta growth factor; PPID: peptidyl-prolyl cis-trans isomerase D; PRL: prolactin; RAN: GTP-binding nuclear protein Ran; SAP: serum amyloid P-component; SPARCL: SPARC-like protein 1; TFF3: trefoil factor 3; TXD12: thioredoxin domain-containing protein 12; XPNPEP1: Xaa-Pro aminopeptidase 1.

The majority of the top-ranked proteins associated with PE were not shared by the two cohorts (Figure 1(A)). In contrast, the majority of the top-ranked proteins associated with GA were shared by the two cohorts (Figure 1(B)). The plasma level of the top-ranked protein included in the model predicting PE in the Stanford cohort only (SPARC-like protein 1), and the Detroit cohort only (MMP7 or matrilysin) are depicted over the course of pregnancy for women with PE and women with uncomplicated pregnancies in Supplementary Figure 4.

### GO analysis

The most significant genes corresponding to the proteins most highly associated with PE (*p* < 1.0E–04) were extracted from both datasets (Supplementary Figure 5). Proteins pointing to inflammatory and immune processes were prominent in the Stanford cohort, while proteins pointing to apoptotic and cell regulatory processes were prominent in the Detroit cohort. The GO analysis indicates that molecular functions and biological processes separating women with PE from women with uncomplicated pregnancies differed between the two cohorts.

## Discussion

Multivariate analyses of large highly multiplexed proteomic datasets revealed highly significant and cross-validated proteomic signatures predicting PE in individual cohorts over the course of a pregnancy. However, these signatures were not generalizable across cohorts. Our results point to a broader issue that is likely relevant to the conduct of proteomic discovery studies in cohorts of patients suffering from a clinical syndrome, such as PE, driven by heterogeneous pathophysiologies. While novel technologies including highly multiplex proteomic arrays and adapted computational algorithms allow for novel discoveries that cross-validate in a particular study cohort, they may not be generalizable. A likely reason is that the prevalence of pathophysiologic processes leading up to the “same” clinical syndrome can be distributed differentially in studied cohorts. As such, signatures derived in individual cohorts may capture different aspects of the pathophysiological spectrum, which is mirrored by different proteomic signatures [11,28]. Our findings indicate the need for studies in diverse and well-phenotyped patient populations that are large enough to carefully characterize subsets of patients with shared pathophysiologies and derive subset-specific proteomic signatures of sufficient predictive power.

The requirement for such studies is reflected by the difficulties to derive sufficiently accurate and clinically useful proteomic signatures for the early prediction of PE [29]. One metric used to assess the performance of classifiers (proteomic signatures) to predict PE is the AUC of receiver operating characteristic (ROC) curves, which depicts the relationship between a classifier’s true- and false-positive rates [30,31]. While some studies report AUCs > 0.8 in specific settings and patient subgroups, the majority of studies report AUCs < 0.8 equating with a fair performance only [5,6,12,32-34].

Single markers including the angiogenic factors sFlt-1 and endoglin, or the ratio between two markers, namely sFlt-1 and PLGF, have received particular attention as predictors of PE [35,36]. While these markers are either significantly elevated or decreased before disease manifestation in a portion of women who later develop PE, they remain in the normal range for a significant fraction of women with PE [32,33,37,38].

A strength and novelty of our study is the combined analysis of two independently collected datasets containing over a thousand simultaneously measured plasma proteins on the same platform. This provided a unique opportunity to examine whether comprehensive proteomic findings inferred in one cohort would generalize in an alternate cohort. Another strength is the derivation of proteomic models for two different clinical endpoints, one physiologic (GA) and the other pathophysiologic (PE) in nature. The divergent findings that proteomic models predicting a physiological pregnancy outcome generalized across the two cohorts, while proteomic models predicting PE did not, strengthen the conclusion that cohort-specific proteomic differences in women with PE likely mirror differences in the predominant underlying pathophysiology. In other words, the proteomic assay performed well enough to infer a generalizable model predicting GA in both cohorts, which makes it less likely that technical aspects of the assay, including batch effects, accounted for observed differences.

Our study has several limitations. The Stanford cohort included women with early- and late-onset PE, while the Detroit cohort included only women with late-onset PE (>34 weeks GA) [12]. While it has been suggested that this dichotomy separates women into two groups with different underlying pathophysiologies, such notion is still subject of ongoing investigations [39,40]. An alternative view is that early- and late-onset PE along with disease severity represent a pathophysiological spectrum with mixed contributions from the placenta and maternal factors that increase susceptibility of the vasculature to damage [11,37,41]. Our cohort size was too small to examine proteomic differences between women with early- and late-onset PE. However, we could address a related question and examine whether the proteomic model derived in the Detroit cohort (late-onset PE) could predict PE in the subset of women in the Stanford cohort with late-onset PE. The fact that such prediction failed supports the view that differential pathophysiological processes, unrelated to the onset of PE, led to development of PE in the two cohorts. The Stanford cohort was heterogeneous with 42% of women being nulliparous, 44% suffering from severe PE, and 89% being Caucasian. Importantly, the Detroit cohort had similar fractions of nulliparous women and women with severe PE. Strikingly different, however, was the racial distribution. Ninety-four percent of women in the Detroit cohort were African American. The possibility that racial differences contributed to the diverse proteomic signatures is intriguing. Racial and ethnic differences in protein signatures associated with PE have previously been reported [42,43]. However, alternative explanations could account for such differences including different environmental conditions, variable healthcare settings, and phenotypical dissimilarities not necessarily captured by the studies.

Our findings have important implications for the design of omic discovery studies for a syndrome like PE. They highlight the need for performing such studies in diverse and well-phenotyped patient populations that are large enough to characterize subsets of patients with shared pathophysiologies to then derive subset-specific signatures of sufficient predictive power.

## Supplementary Material

Supplemental Material

## Figures and Tables

**Table 1.** Patient and pregnancy data.

	Stanford cohort		Detroit cohort	
Demographics	Preeclampsia (*n* = 18)	Controls (*n* = 18)	Preeclampsia (*n* = 76)	Controls (*n* = 90)
Age (years)	32.0 [27.3–37.5]	30.0 [29.0–33.5]	22.0 [21.0–29.0]	24.0 [21.0–27.8]
Body mass index (kg/m^2^)	27.9 [22.3–31.3]	22.3 [19.9–23.7]	30.0 [24.8.0–36.2]	26.5 [22.8–33.2]
Race				
African American	1	0	72	84
Asian	2	0	0	0
Caucasian	14	18	4	6
Unknown	1	0	0	0
Pregnancy				
Gravity	2 [1–2]	2 [1–3]	2 [1–4]	3 [2–5]
Parity (nulliparity)	7	8	32	26
Preeclampsia (mild/severe)				
Early preeclampsia	3/2			
Late preeclampsia	5/8		48/28	
Gestational age at delivery				
Early preeclampsia	31.3 [30.1–32.1]			
Late preeclampsia	38.3 [37.1–39.6]		38.7 [37.7–39.4]	
All		39.8 [39.1 −40.8]		39.4 [39.0–40.4]

Reported are the median and interquartile range or absolute numbers.
