# Supplementary material for: Proteomic signatures predict preeclampsia in individual cohorts but not across cohorts – implications for clinical biomarker studies
Source: J Matern Fetal Neonatal Med. Author manuscript; Available in PMC 2022 Dec 1. (PMC8410912; doi:10.1080/14767058.2021.1888915)
Supplement: Supplemental Material [file NIHMS1730051-supplement-Supplemental_Material.docx]

**Supplementary Methods**

*Derivation of a proteomic model predicting PE in Stanford cohort:* For a matrix *X* containing all proteins (features), and a binary vector of preeclampsia Y, a multivariate logistic regression model with penalization (LASSO) was developed to calculate the coefficients *β* for each entity in *X* to maximize the overall log-likelihood using the conditional likelihood:

$\mathcal{l}\left( \beta\right)=\sum_{i=1}^{n} \log p_{y_{i}}\left( x_{i};\beta) \right.$, (1)

where $p_{y_{i}}\left( x_{i};\beta\right)=Pr(C=y_{i}|X=x_{i};\beta)$. The new log-likelihood can be rewritten as:

$\mathcal{l}\left( \beta\right)=\sum_{i=1}^{n} \left[ y_{i}\beta^{T}x_{i}- \log\left( 1+e^{\beta^{T}x_{i}} \right) \right].$ (2)

A $L_{1}$ regularization was applied on the *λ* coefficient to reduce the model complexity, such that:

$\mathcal{l}\left( \beta\right)=\sum_{i=1}^{n} \left[ y_{i}\beta^{T}x_{i}- \log\left( 1+e^{\beta^{T}x_{i}} \right) \right]+\lambda\sum_{j=1}^{p} \left| \beta_{j} \right|$, (3)

where $\lambda$ is selected by cross-validation.

**Supplementary Figure 1**

**
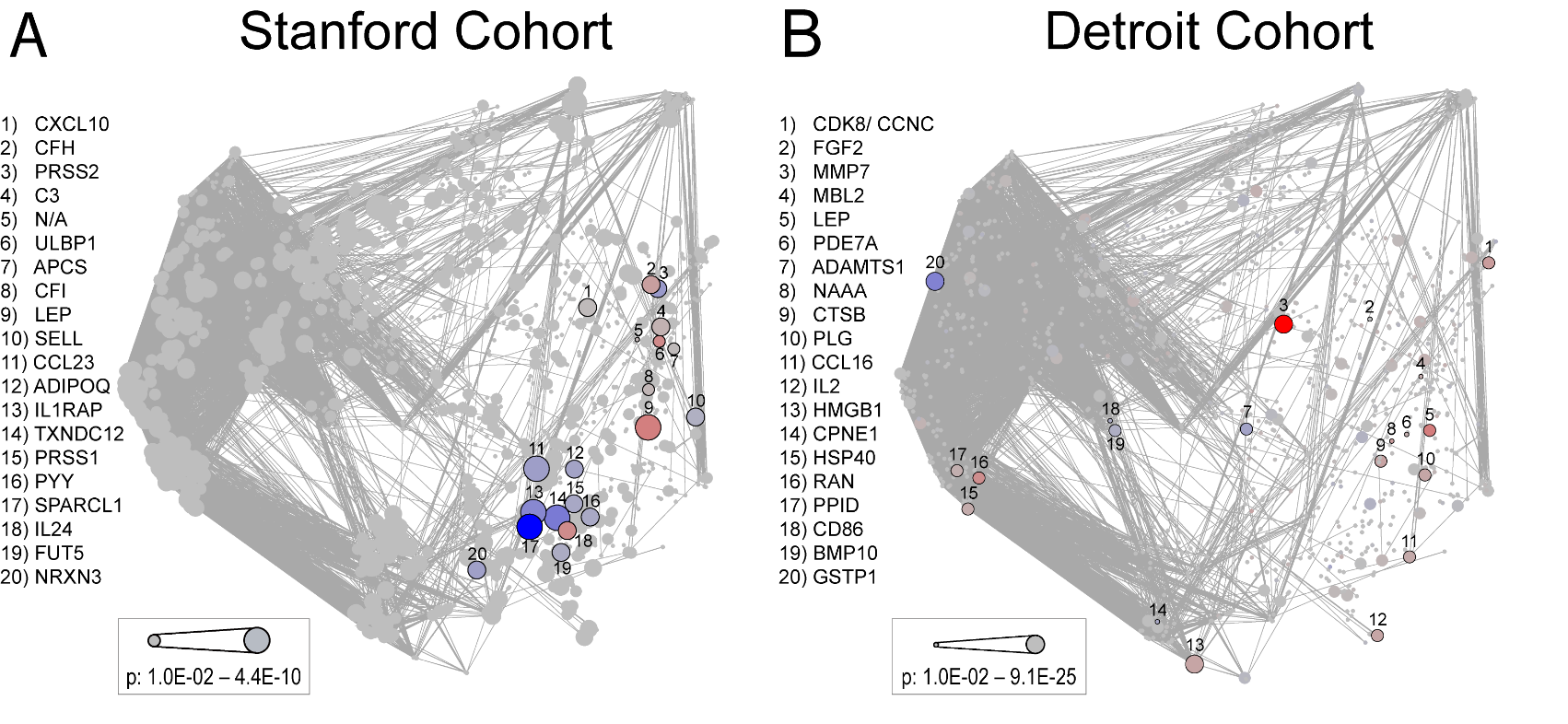
**

**Separate proteomic models predict PE in the two cohorts**

**(A)** The top 20 proteins contained in the model predicting PE in the Stanford cohort are listed by gene symbols and are projected onto the correlation network featuring all 1,116 measured proteins. Model proteins are color-coded, red and blue indicating positive and negative correlations, respectively. The dot size is proportional to the p-value, while the color intensity is proportional to the correlation coefficient. Gray lines indicate significant correlations between proteins (p = 1.0 E-37, Bonferroni corrected and further adjusted for sparse representation). **(B)** The top 20 proteins contained in the model predicting PE in the Detroit cohort are listed by gene symbols and are projected onto the correlation network. Visual inspection reveals that different proteins predicted PE in the two cohorts.

**Supplementary Figure 2**

**Proteomic models predicting PE are not generalizable across cohorts**

The scatter plots depict the performance of respective proteomic models over the course of pregnancy. The numerical value of the model parameter predicting PE for each woman at the time of sampling (GA) is shown as a color-coded circle. The model parameters (red circles) strongly differentiated women with PE from women with uncomplicated pregnancy (blue circles). Color-coded lines reflect the moving averages. P-values and AUCs of the ROC curves are shown in the left upper corner of each plot. A highly significant proteomic model separating women with PE from women with uncomplicated pregnancies was derived in the Stanford cohort. **(B)** However, this model did not separate women with PE from women with uncomplicated pregnancies in the Detroit cohort as indicated by largely overlapping model parameters. **(C)** Similarly, A highly significant proteomic model fairly separating women with PE from women with uncomplicated pregnancies was derived in the Detroit cohort. **(D)** This model did not separate women with PE from women with uncomplicated pregnancies in the Stanford cohort as indicated by largely overlapping model parameters.

**Supplementary Figure 3**

**Proteomic models predicting GA generalize across cohorts**

**(A)** The scatter plots depict GA *versus* model values (GA prediction) for each women and sampling time in the Stanford cohort. The model accurately predicted GA as indicated by the regression line and adjacent 95% confidence intervals (red lines). **(B)** The proteomic model derived in the Stanford cohort also predicted GA in the Detroit cohort. **(C)** The scatter plots depict GA *versus* model values in the Detroit cohort. The model accurately predicted GA. **(D)** The proteomic model derived in the Detroit cohort also predicted GA in the Stanford cohort.

**Supplementary Figure 4**


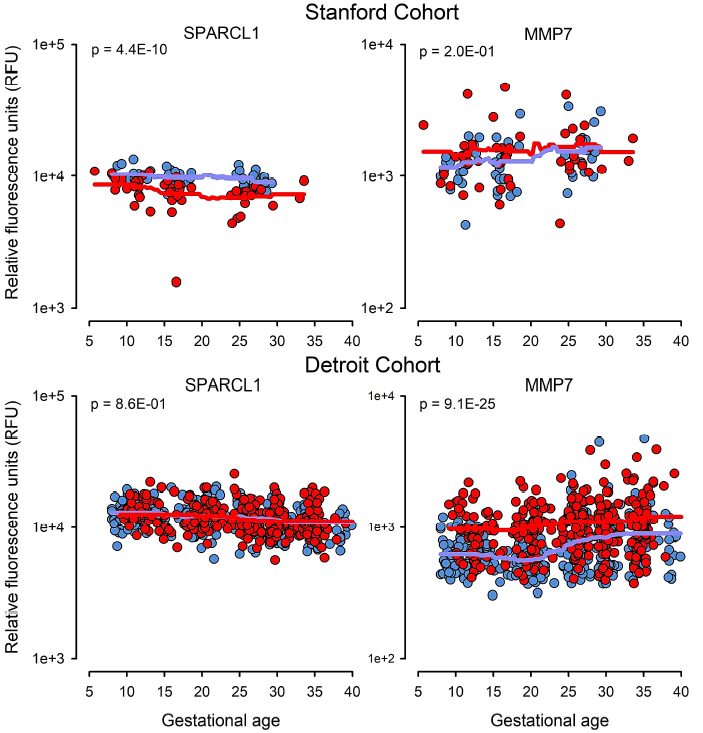


**Plasma levels of the top ranking model protein in the Stanford and Detroit cohort**

Depicted are SPARCL1 and MMP 7. SPARCL1 was the top ranking model protein separating women with PE (red dots) from women with uncomplicated pregnancies (blue dots) in the Stanford cohort. It did not separate women in the Detroit cohort. MMP7 was the top ranking model protein separating women with PE from women with uncomplicated pregnancies in the Detroit cohort. It did not separate women in the Stanford cohort. MMP7, Matrilysin; SPARCL, SPARC-like protein 1.

**Supplementary Figure 5**


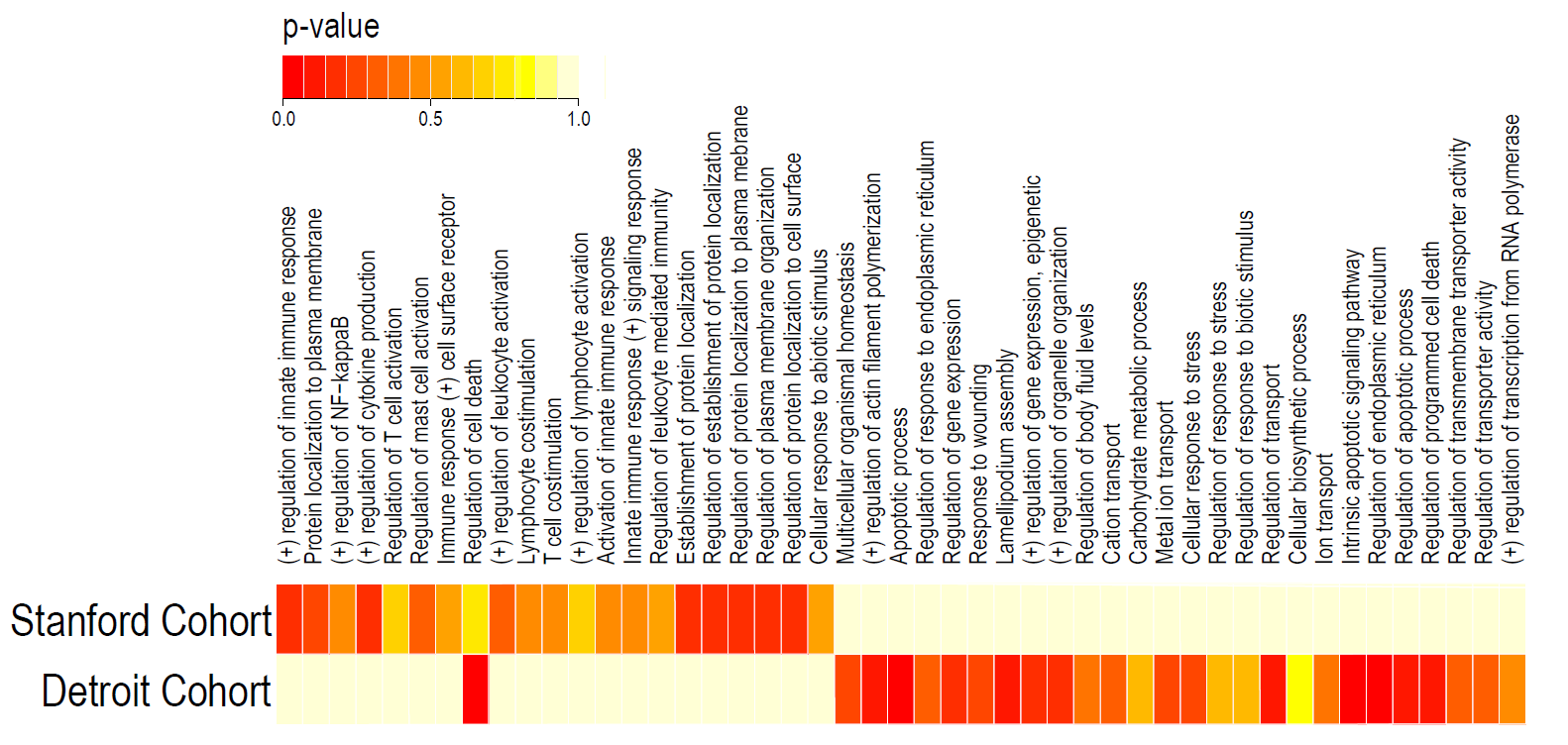


**Biological processes associated with PE differ between cohorts**

The heat map depicts results of the GO analysis. The color code on top reflects p-values associated with individual GO terms for the Stanford and Detroit cohorts. GO terms reaching significance in the two cohorts were distinctly different and pointed to immune and inflammatory processes in the Stanford cohort, and apoptotic and cell-regulatory processes in the Detroit cohort.
